# Double Trouble: Combined Prekallikrein and IgA Deficiencies in a Patient Undergoing Orthotopic Heart Transplantation—A Case Report

**DOI:** 10.1155/cria/9463500

**Published:** 2026-04-27

**Authors:** Brittany McLay, Jocelyn E. Coholich, Katelyn Glines, Jonathan A. Bond, Mir Ali Abbas Khan, Brian P. Peppers, Sonikpreet Aulakh

**Affiliations:** ^1^ Department of Anesthesiology, West Virginia University, Morgantown, West Virginia, USA, wvu.edu; ^2^ West Virginia University School of Medicine, Charleston, West Virginia, USA, wvu.edu; ^3^ Department of Anesthesiology, Southern Oregon Anesthesia, Medford, Oregon, USA, oregon.gov; ^4^ Division of Cardiac and Thoracic Anesthesiology, Department of Anesthesiology, West Virginia University, Morgantown, West Virginia, USA, wvu.edu; ^5^ Department of Adult and Pediatric Allergy/Immunology, West Virginia University, Morgantown, West Virginia, USA, wvu.edu; ^6^ Department of Medical Oncology, West Virginia University, Morgantown, West Virginia, USA, wvu.edu

**Keywords:** cardiac transplantation, case report, IgA deficiency, prekallikrein deficiency, transfusion

## Abstract

Prekallikrein (PK) and selective IgA deficiencies are rare, and their coexistence in a cardiac transplant patient presents unique challenges. These disorders affect anticoagulation monitoring and transfusion safety, necessitating tailored perioperative strategies. We present the case of a 58‐year‐old female with both PK and IgA deficiency who underwent orthotopic heart transplantation (OHT), complicated by significant postoperative bleeding requiring re‐exploration. A multidisciplinary plan included FFP‐based IgA desensitization and correction of elevated ACT from PK deficiency. Anti‐Xa levels were used to confirm anticoagulation. The patient tolerated unwashed blood products without anaphylaxis and recovered uneventfully. This is the first known report of combined PK and IgA deficiencies in cardiac surgery. It highlights a successful strategy involving perioperative FFP administration for both desensitization and ACT normalization. These measures ensured safe anticoagulation and transfusion in a high‐risk setting.

## 1. Introduction

In cardiac transplantation, encountering patients with rare coexisting disorders requires careful management, especially given emergent time constraints. Here, we present the case of a patient undergoing orthotopic heart transplantation (OHT) with selective IgA deficiency and prekallikrein (PK) deficiency, highlighting the complex challenges of these conditions in the perioperative period.

Selective IgA deficiency is the most common primary immunodeficiency, characterized by undetectable serum IgA levels with normal IgG and IgM. While many individuals remain asymptomatic, approximately one‐third of patients with IgA deficiency develop severe transfusion reactions, including anaphylaxis, if exposed to blood products containing IgA from normal donors [1]. While the risk can be mitigated by blood product washing, this process can cause a theoretical risk of bacterial infection and a reduction in red blood cell (RBC) content; however, this has not been shown to be clinically significant. Thus, IgA deficiency adds an additional layer of complexity to transfusion management, given the heightened concern for blood product requirements following transplantation [2].

PK deficiency, also known as Fletcher factor deficiency, is a rare autosomal recessive disease characterized by a prolonged activated partial thromboplastin time (aPTT) with normal prothrombin time (PT) and increased activated clotting time (ACT). Although laboratory abnormalities exist, PK deficiency is not associated with bleeding diathesis [3]. Merely 80 PK cases are documented within the medical literature, and its impact on cardiac surgery remains poorly understood [4]. A major concern in these patients is the ability to safely and accurately anticoagulate during cardiopulmonary bypass (CPB) given the known disruptions in the coagulation cascade. Prolonged aPTT and ACT can make conventional anticoagulation monitoring inaccurate or unreliable, creating significant challenges in CPB perioperative management.

This report explores the perioperative considerations required to optimize outcomes in cardiac transplant patients with rare coexisting medical conditions. The simultaneous presence of both selective IgA deficiency and PK deficiency in a patient undergoing OHT is exceptionally rare and presents unique challenges. A PubMed search (February 2026) found no cases of combined PK and IgA deficiency in cardiac surgery, highlighting the report’s novelty. The objective of this case report is to examine the distinct perioperative management strategies employed and to review the relevant literature to guide future care for similarly complex patients.

## 2. Case Presentation

**FIGURE 1:**
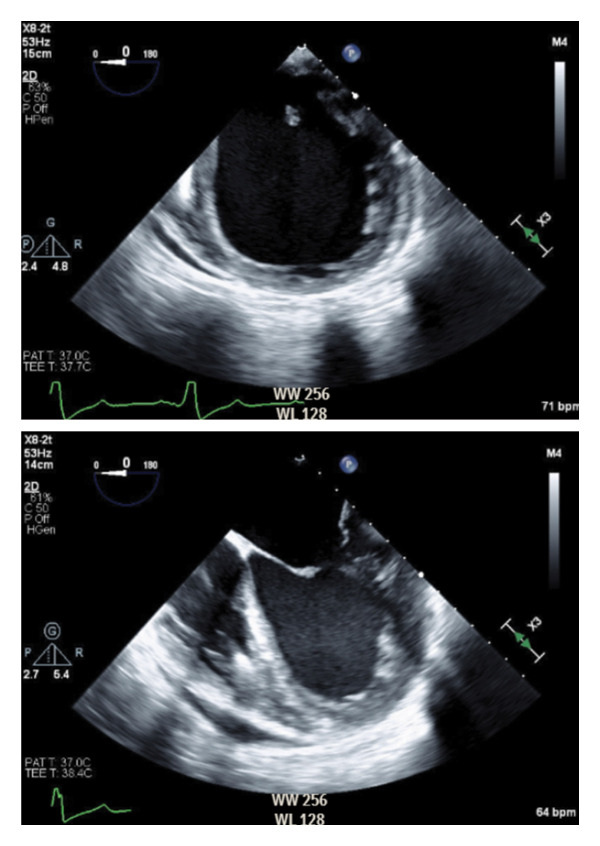
Transesophageal echocardiography images prior to orthotopic heart transplantation displaying a severely dilated left ventricle with severely reduced left ventricular systolic function.

A 58‐year‐old female with a past medical history of heart failure with reduced ejection fraction secondary to familial non‐ischemic cardiomyopathy, selective IgA deficiency, PK deficiency, paroxysmal atrial fibrillation, obstructive sleep apnea on continuous positive airway pressure, obesity status post bariatric surgery, noninsulin‐dependent Type II diabetes mellitus, peripheral vascular disease, hypertension, and hyperlipidemia presented for OHT following several recent admissions for heart failure exacerbations. Preadmission transthoracic echocardiogram revealed a severely dilated left ventricle with eccentric hypertrophy and severely reduced ejection fraction of 27.9% (Figure 1). Additional findings included abnormal left ventricular diastolic function with elevated filling pressures, severely dilated right ventricle with mildly depressed function, right ventricular systolic pressure consistent with severe pulmonary hypertension, and severe mitral regurgitation. The patient subsequently underwent intra‐aortic balloon pump (IABP) insertion via the right femoral arterial approach as a bridge to transplant. The right femoral artery balloon pump was later removed and replaced with an IABP via the left axillary artery to facilitate ambulation.

Pretransplantation testing confirmed an IgA level of < 3(mg/dL), with normal levels of IgG, consistent with a diagnosis of IgA deficiency [5] (Table 1). The patient had no prior history of blood transfusion. Additionally, lab testing revealed prolonged aPTT with normal PT values, leading to the diagnosis of PK deficiency (Table 1). Prior to transplantation, hematology and immunology were consulted to create a perioperative management plan in conjunction with the transplantation, cardiac surgery, and cardiac anesthesiology teams.

**TABLE 1:** Admission laboratory results with corresponding reference ranges.

Results	Patient value	Reference range
Immunoglobulin A	< 3	85–499 mg/dL
Immunoglobulin G	1877	610–1616 mg/dL
Anti‐IgA Antibody	< 99	< 99 U/mL
Prothrombin Time (PT)	12.9	9.1–13.9 s
Activated Partial Thromboplastin Time (aPTT)	71.6	24.2–37.5 s

**FIGURE 2:**
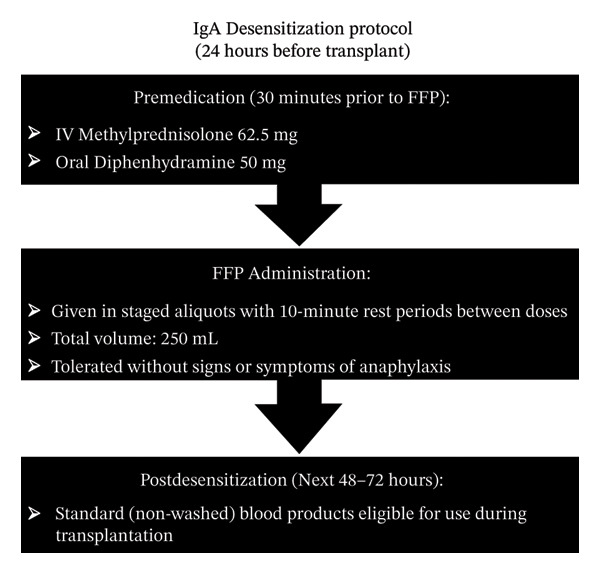
IgA Desensitization Protocol. Timeline of premedication (methylprednisolone 62.5 mg IV, diphenhydramine 50 mg oral) and FFP administration (50 cc, 100 cc, 100 cc over 1 h with 10‐min intervals) 24 h pretransplant.

Approximately 24 h before the scheduled transplant date, the patient underwent an IgA deficiency desensitization protocol using fresh frozen plasma (FFP) (Figure 2). Premedication included intravenous (IV) methylprednisolone (62.5 mg) and oral diphenhydramine (50 mg) administered 30 min before initiation. FFP was administered in escalating doses (50 cc, 100 cc, 100 cc) over 1 h, totaling 250 cc, with 10‐min intervals. The patient tolerated the transfusion without incident, allowing the use of standard (nonwashed) blood products for 48–72 h after desensitization. On the morning of the procedure, the patient’s aPTT was 47 (sec), and ACT was 195 (sec) (Table 2). To correct the patient’s ACT prior to transplant, 2 additional units of FFP were transfused 30min before the operating room time and were completed prior to induction of anesthesia. Following FFP administration, the aPTT improved to 36.9 (sec), ACT to 99 (sec), and the anti‐Xa level was < 0.06 (units/mL) (Table 2).

**TABLE 2:** Preoperative laboratory results with corresponding reference ranges.

Results	Patient value	Reference range
*(Lab values approximately 6 h before surgery)*		

Activated Clotting Time	195	70–120 s
Activated Partial Thromboplastin Time (aPTT)	47	24.2–37.5 s

**2 additional units of FFP administered for correction prior to OR**		

*(Lab values approximately 30 min before surgery)*		

Activated Clotting Time	99	70–120 s
Activated Partial Thromboplastin Time (aPTT)	36.9	24.2–37.5 s
Anti‐Xa	< 0.06	< 0.06 IU/mL

**FIGURE 3:**
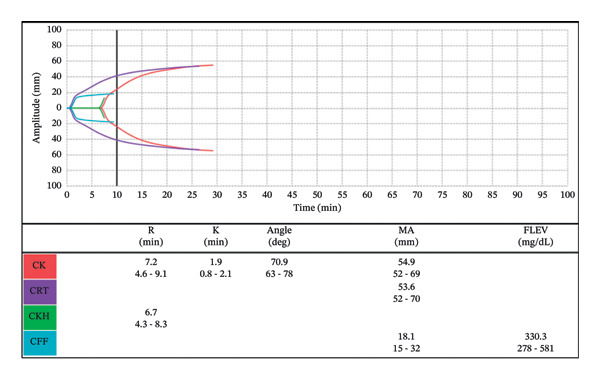
Postoperative TEG displaying no active coagulopathy.

The patient underwent a heart transplant from a brain dead, beating heart donor. A standard 24,000 unit heparin dose was utilized for CPB, and aminocaproic acid (antifibrinolytic) was administered per department protocol. Values for ACT and anti‐Xa levels 3  min post heparin were 562 (sec) and > 2.0 (units/mL), respectively. Subsequent values throughout the CPB revealed adequate ACT values ranging from 479–562 (sec), with corresponding levels of anti‐Xa of > 2.0 (units/mL) (Table 3). After weaning from CPB, protamine was administered, resulting in an ACT of 103 (sec) and anti‐Xa of < 0.06 (units/mL). Autologous blood transfusion was utilized throughout the case, with the patient receiving a total of 455 cc after CBP. Thromboelastography (TEG) postprotamine did not display coagulopathy, and no additional blood products were required intraoperatively (Figure 3). She remained intubated on inhaled nitric oxide and a milrinone infusion for right ventricular dysfunction and was transported to the cardiovascular intensive care unit (CVICU).

**TABLE 3:** Intraoperative ACT and anti‐Xa monitoring.

Time	08:58	09:10	Surgery start	10:11	10:21	10:40	10:51	11:04	11:17	11:27	11:47	11:52	Surgery stop	12:09	13:00
ACT (sec)	99^∗^	—		449	562	534	543	—	479	515	503	—		103^∗∗^	—
Anti‐Xa (units/mL)	—	< 0.06^∗^		—	—	—	—	> 2	—	—	—	> 2		—	< 0.06^∗∗^

^∗^Baseline values.

^∗∗^Postprotamine values.

**FIGURE 4:**
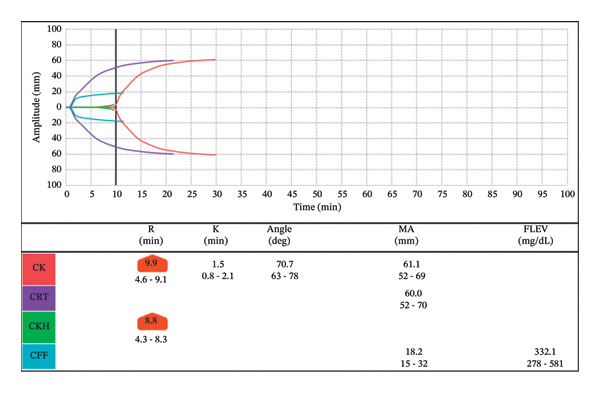
TEG drawn following increased chest tube output displaying coagulopathy, treated with FFP.

**FIGURE 5:**
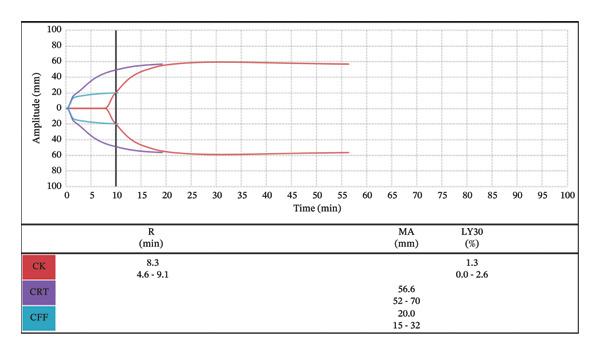
Postblood product administration TEG displaying correction of the coagulopathy with ongoing bleeding pending return to OR.

Postoperatively, the patient developed significant bleeding and large volume output from the surgical chest tubes. A complete blood count (CBC) and TEG were drawn to help guide transfusion in which the patient received 2 units of packed RBCs (PRBCs) and 4 units of FFP for a prolonged *R* time (Figure 4). Despite correction of the coagulopathy (Figure 5), 1 unit of platelets and 10 pooled units of cryoprecipitate, all unwashed products, were administered due to ongoing bleeding. The chest tube output continued to rise, and the patient returned to the operating room for surgical re‐exploration. During this procedure, she received 3 units of PRBCs, 2 units of FFP, and 1 unit of platelets, along with 500 cc of autologous blood. The cardiac surgery team identified the surgical bleed, and the patient returned to the CVICU intubated. The patient was extubated later that day to high‐flow nasal cannula. At no point did she exhibit signs of anaphylaxis or hemodynamic instability following any of the blood transfusions.

## 3. Discussion

Our patient underwent routine pretransplant screening which revealed low levels of IgA. Clinical IgA deficiency is defined by decreased IgA levels (< 7 mg/dL) and normal IgG and IgM levels [5]. The prevalence of IgA deficiency ranges from 1:143 to 1:965, with equal distribution between both genders [5]. Anti‐IgA antibodies have been found in 20%–40% of IgA‐deficient patients, with an incidence of anaphylaxis to blood products ranging from 1:20,000 to 1:47,000 [5]. Our patient had no prior transfusions and had not been previously screened for IgA deficiency.

To minimize the risk of anaphylactic blood transfusion reaction associated with IgA deficiency, the patient underwent a desensitization protocol using FFP 24 h before the scheduled transplant time. The rationale behind this desensitization approach is that gradual exposure to small amounts of donor IgA may attenuate the immune response in patients with anti‐IgA antibodies, thereby reducing the likelihood of transfusion‐related anaphylaxis. Other strategies, such as the use of washed or IgA‐deficient blood products, pretransfusion IgA antibody quantification, or immunoglobulin concentrate (e.g., Pentaglobin and Gammagard) desensitization, have also been described; however, these options are often logistically challenging and impractical in the urgent setting of transplantation. Although data remain limited, a few isolated case reports have documented successful use of FFP or immunoglobulin concentrate‐based desensitization, supporting its feasibility when alternative approaches are not readily available. A 19‐year‐old undergoing liver transplantation had a history of severe anaphylactic reaction to RBCs, cryoprecipitate, and platelets. She received Pentaglobin (IgM‐enriched immunoglobulin), which allowed for the administration of unwashed blood products for the transplantation [1]. An additional case report also described a 6‐year‐old male with common variable immunodeficiency who had previous anaphylaxis with IV immunoglobulin (IVIG). He safely received Gammagard (immune globulin infusion) and tolerated future treatments well [6].

In previous reports, the alternative approach of product washing requires extensive logistics to maintain viable IgA‐deficient products, including platelets with a shelf life of only 5  days [7]. Washed blood products are frequently impractical to obtain in the urgent context of cardiac transplantation, where timing is critical and coordination with blood banks can introduce significant delays. This limitation extends beyond the intraoperative period, as patients may require immediate access to blood products for management of acute postoperative bleeding, as occurred in our case, making the use of washed or IgA‐deficient units logistically unfeasible. In addition to the difficulty of obtaining sufficient products from IgA‐deficient patients and the short shelf life of washed blood, cardiac transplant has the potential for large‐volume resuscitation. The use of blood products during cardiac transplantation is extremely common, with a recent study reporting an average use of 7.83 units of PRBCs per patient [8]. The paper written by Kiani‐Alikhan suggested that in patients with a history of IgA deficiency, desensitization protocols should be considered if there is potential for the administration of blood products as an emergency, lifesaving treatment [1]. The desensitization approach allows for the safe use of unwashed blood products for up to 48–72 h. Alternatives to FFP‐based desensitization in IgA deficiency include pretransfusion quantification of anti‐IgA antibodies or the use of immunoglobulin concentrates for desensitization; however, both approaches have limitations. Quantifying IgA antibodies may not eliminate the risk of anaphylaxis, as even patients without detectable antibodies can react to unwashed blood products, and the added risk is particularly high in urgent cardiac transplantation, where hemodynamic instability is common. Immunoglobulin concentrates, while potentially effective, are often difficult to obtain rapidly in urgent settings and can be costly, limiting their practicality during emergent transplantation.

Although our patient only required autologous blood transfusion administration during the initial intraoperative course, she developed acute postoperative bleeding that required exploration in the OR. At that time, she was given unwashed blood products to help with the acute resuscitation. She had no signs of anaphylactic reaction following administration of the blood products.

Selective IgA deficiency also carries an increased risk of recurrent infections, particularly of the sinopulmonary and gastrointestinal tracts, reflecting the important role of IgA at mucosal surfaces; this risk becomes especially relevant after transplantation when immunosuppressive therapy further compromises host defense. Awareness of this is critical for postoperative monitoring, timely initiation of infection prophylaxis, including vaccines and possibly antimicrobial strategies, and increased awareness for early signs of infection in the immunocompromised setting [9].

PK deficiency was also discovered during routine lab testing for the transplant evaluation. Patients with PK deficiency have baseline elevations in their ACT levels as the ACT assay relies on endogenous factors from the kallikrein–kinin system to activate the coagulation pathway [4]. PK deficiency should be evaluated in asymptomatic patients with prolonged aPTT that corrects on incubation, alongside normal levels of contact activation factors and factor inhibitors [3]. Although there are abnormalities in the coagulation cascade, these have not been shown to cause clinically significant bleeding. Since ACT is the primary method for monitoring anticoagulation during cardiac bypass, baseline elevations can raise concerns about whether adequate anticoagulation has been achieved for the conduct of CPB.

In our case, preoperative FFP administration was used to optimize coagulation parameters, aligning with limited reports describing similar management strategies in PK deficiency. In a previous case report involving a 15‐year‐old with PK deficiency undergoing open atrial septal defect repair, FFP was administered at 15 cc/kg over an hour, which normalized ACT levels prior to surgery. This allowed for monitoring of anticoagulation using ACT after the administration of heparin and throughout CPB [4]. An additional case report describes a 6‐year‐old patient undergoing atrial septal defect closure with CPB. This patient received a bolus of FFP at 10 cc/kg, followed by a continuous infusion at 5 cc/kg/hr. Anticoagulation was monitored intraoperatively using both ACT and TEG. The procedure was completed successfully with adequate anticoagulation, and no bleeding complications were observed following separation from bypass [10].

Since adequate anticoagulation is imperative during CPB, we opted to trend anti‐Xa levels along with ACT as a safeguard throughout the case (Table 1). Studies have shown that in severe PK deficiency, anti‐Xa levels can be used as a reliable measure of anticoagulation when high doses of heparin are required to initiate CPB [11]. In this case, anti‐Xa correlated with the ACT throughout the entirety of CPB, including postheparin reversal with protamine. However, a key limitation was the 30–40 min turnaround time for anti‐Xa testing. This delay could produce major issues in the setting of inadequate anticoagulation during CPB and potential clotting of the circuit. Point‐of‐care anti‐Xa testing, if available, could reduce delays, enhancing real‐time anticoagulation monitoring. Despite this, the ACT remained reliable after preoperative correction. An alternative strategy for monitoring adequate anticoagulation during CPB in patients with PK deficiency is the use of point‐of‐care Hepcon‐based heparin concentration monitoring, as described by Cankovic et al. This method employs a heparin–protamine titration technique to directly quantify circulating heparin levels, providing a reliable measure of anticoagulation throughout CPB. By avoiding reliance on contact protein‐dependent reagents, this approach overcomes the limitations associated with ACT–based monitoring [12].

In summary, this case illustrates the successful multidisciplinary management of a patient with both PK and IgA deficiencies undergoing cardiac transplantation. For IgA deficiency, the use of FFP for desensitization allowed for the safe administration of unwashed blood products during the perioperative period, as our patient experienced acute postoperative bleeding that required life‐saving blood products. Regarding PK deficiency, FFP administration effectively restored ACT levels for safe CPB management. Additionally, anti‐Xa levels, although delayed compared to ACT, confirmed adequate anticoagulation and its reversal following completion of the transplant.

## 4. Conclusion

This case, the first reported of combined PK and IgA deficiencies in OHT, demonstrates the efficacy of FFP‐based desensitization for IgA deficiency and preoperative FFP for PK deficiency to ensure safe transfusion and adequate anticoagulation during CPB. These strategies facilitated uneventful OHT despite postoperative bleeding requiring re‐exploration. Limitations include delayed anti‐Xa results, highlighting the need for point‐of‐care testing. Clinicians should consider similar protocols for patients with rare coagulopathies and immunodeficiencies undergoing high‐risk surgeries. Prospective studies are needed to validate these approaches and optimize perioperative management.

## Author Contributions

Brittany McLay, contributed to the intraoperative anesthetic care of the patient, was involved with the literature review, formulation of the publication as well as the submission.

Jocelyn E. Coholich was involved with the literature review and formulation of the publication.

Katelyn Glines contributed to the intraoperative anesthetic care of the patient and was involved with formulation of the publication.

Jonathan A. Bond contributed to the intraoperative anesthetic care of the patient and was involved with the formulation of the publication.

Mir Ali Abbas Khan contributed to the intraoperative anesthetic care of the patient and formulation of the publication.

Brian P. Peppers contributed to the preoperative and postoperative care for this patient as the consultant immunologist as well as formulation of the publication.

Sonikpreet Aulakh contributed to the preoperative and postoperative care for this patient as the consultant hematologist as well as formulation of the publication.

## Funding

No funding was received for this research.

## Consent

Written consent was obtained from the patient for the publication of this case report.

## Conflicts of Interest

The authors declare no conflicts of interest.

## Data Availability

Data are included throughout this case report to provide support for the conclusions. Further data inquiries are at the discretion of the corresponding author with consideration for institutional policy and protection of patient confidentiality.
